# Full gaze contingency provides better reading performance than head steering alone in a simulation of prosthetic vision

**DOI:** 10.1038/s41598-021-86996-4

**Published:** 2021-05-27

**Authors:** Nadia Paraskevoudi, John S. Pezaris

**Affiliations:** 1 Brainlab - Cognitive Neuroscience Research Group, Department of Clinical Psychology and Psychobiology, University of Barcelona, Barcelona, Spain; 2 Institute of Neurosciences, University of Barcelona, Barcelona, Spain; 3 Department of Neurosurgery, Massachusetts General Hospital, Boston, MA USA; 4 Department of Neurosurgery, Harvard Medical School, Boston, MA USA

**Keywords:** Perception, Sensory processing, Reading, Translational research, Vision disorders

## Abstract

The visual pathway is retinotopically organized and sensitive to gaze position, leading us to hypothesize that subjects using visual prostheses incorporating eye position would perform better on perceptual tasks than with devices that are merely head-steered. We had sighted subjects read sentences from the MNREAD corpus through a simulation of artificial vision under conditions of full gaze compensation, and head-steered viewing. With 2000 simulated phosphenes, subjects (*n* = 23) were immediately able to read under full gaze compensation and were assessed at an equivalent visual acuity of 1.0 logMAR, but were nearly unable to perform the task under head-steered viewing. At the largest font size tested, 1.4 logMAR, subjects read at 59 WPM (50% of normal speed) with 100% accuracy under the full-gaze condition, but at 0.7 WPM (under 1% of normal) with below 15% accuracy under head-steering. We conclude that gaze-compensated prostheses are likely to produce considerably better patient outcomes than those not incorporating eye movements.

## Introduction

Visual scanning is an inherently automatic aspect of perceiving our external world, and supporting these motions in artificial vision devices has been one of many challenges for the field of visual prostheses. Visual prosthesis devices to restore function to the blind have been under investigation and development for nearly a century (for reviews see^1–6^), with the most recent major milestones being approval of devices for clinical use in the US and Europe (Alpha IMS and AMS, Retina Implant AG, Reutlingen, Germany; Argus II, Second Sight Medical Products Inc, Sylmar, CA; PRIMA and IRIS II, Pixium Vision, Paris, France^1, 7–13^). To-date, most visual prostheses provide a modest level of visual function through electrical stimulation applied to a collection of electrode contacts at one of the stages of the early visual pathway (e.g., retina, optic nerve, lateral geniculate nucleus or primary visual cortex). Each of these contacts is used to stimulate a small volume of neural tissue and evoke a focal visual percept called a *phosphene*, colloquially known as the pixels of artificial vision. Coordinated stimulation across electrodes creates patterns of phosphenes through which images are conveyed to the subject, providing restoration of function.

Visual prostheses can be classified into two broad categories, those that use the optics of the eye as a critical part of their design, typically placing photodiode arrays on the retina that turn light focused by the eye into patterns of neural activity (Alpha IMS and AMS, PRIMA), and those that deliver stimulation without employing the optics of the eye (Argus II, Orion, EPIRET3, IRIS II, Boston Retinal Implant, Australian bionic eye, CORTIVIS, etc.^1, 14–20^; see reviews by Lews and colleagues^21^ and Yue and colleages^22^). For subjects to explore the visual scene, these two classes of devices present very different interfaces. The first group employs the natural optics and is *gaze steered*, that is the direction of interest is controlled by the subject through both eye and head movements, while the second group does not employ the natural optics and is *head steered*, that is, the direction of interest is controlled only by head position*.* Here, we will be concerned only with the latter group.

Head-steered visual prostheses use a camera to image the external world, translating signals from that camera into patterns of neural stimulation that are delivered to the early visual pathway without engaging the optics of the eyes^5, 6^. Most often the cameras in these devices are mounted on a set of goggles, roughly aligned with the optical axis of the eyes. To emphasize the distinction from gaze-steered prostheses, head-steered prostheses have an important limitation that they are controlled only through motions of the head and do not incorporate eye position information. It is this specific issue we address with the present report.

The influence of oculomotor processes such as saccadic movements on normal visual perception is substantial (see review by Paraskevoudi and Pezaris^23^). Eye movements used to scan our environment cause the visual presentation of external objects to move across our retinas^24–26^. Despite these frequent displacements, the brain constructs a stable representation of our surroundings by compensating each moment’s retinal snapshot with information about the corresponding gaze position^5, 26, 27^ through a process called *spatial updating*. In diseases such as oscillopsia^28^ where the brain no longer has a accurate representation of the position of the eye^29^, the visual and cognitive effects can be debilitating^30^, because spatial updating can no longer work properly. In normal visual perception, even small eye movements are important as they modulate visual processing^31–33^, optimize eye position during fixation^32, 34^, and prevent retinal fading^35^. Thus, eye movements and spatial updating contribute to the correct localization and perception of objects, making them critical for visuomotor coordination, navigation, and interaction with the external world.

**Figure 1:**
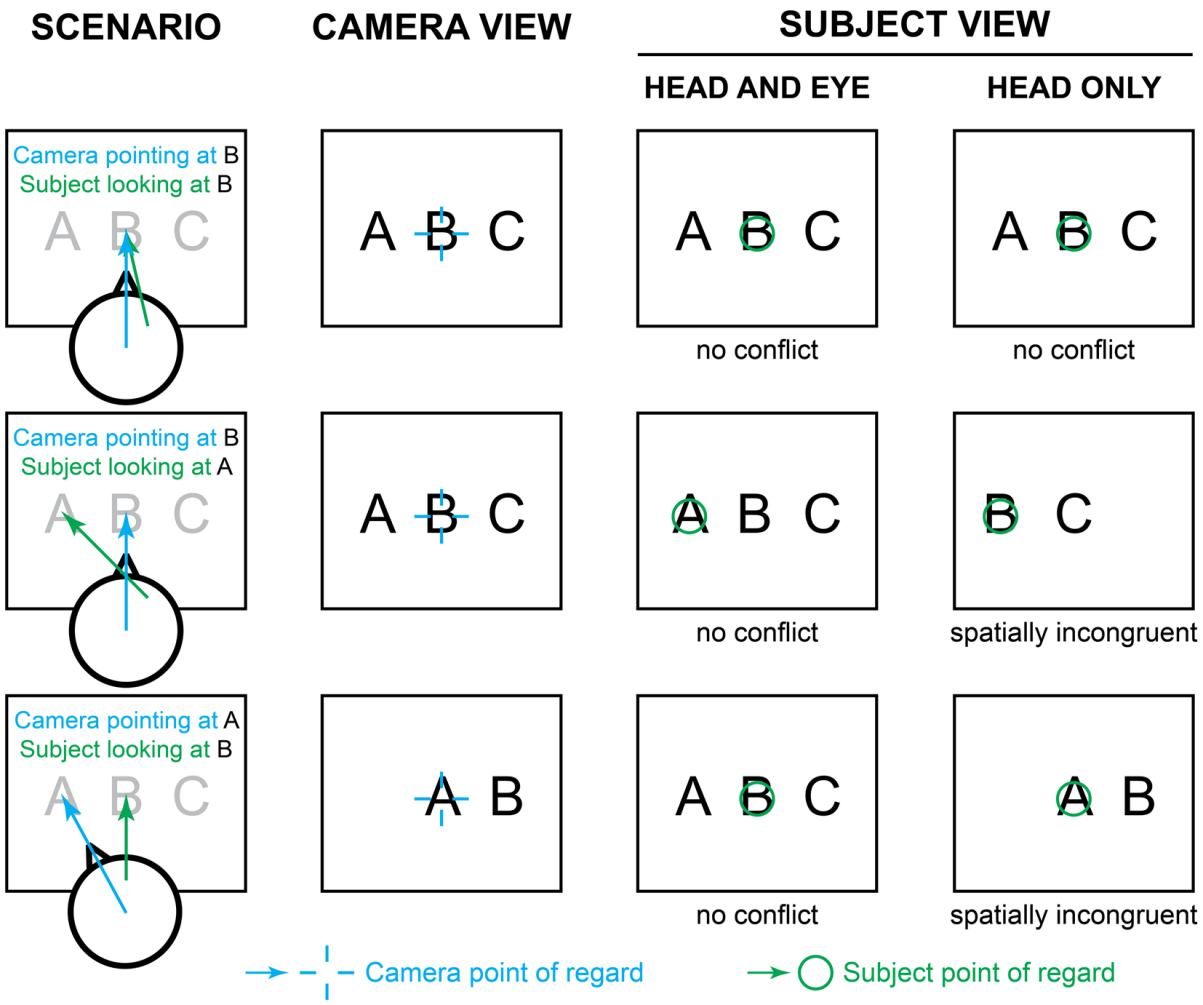
Gaze conditions and the problem created by ignoring eye position. In the general case where the eyes are not used to image the external world in a visual prosthesis, there is potential for the camera axis, typically steered by head position, and the gaze location, determined by the combination of head position and eye position within the head, to be out of agreement. This figure depicts three conditions, by row, the first where the gaze (blue) and camera (green) are aligned, pointing forward (top row), the second where the head and camera are in the same positions, but the eyes have deviated to the left (middle row), and the third where the head and camera have been turned to the left, but the eyes have counter-rotated to the right such that gaze is once again straight ahead (bottom row). The second column from the left shows the image that the camera sees. The third column depicts what is perceived when the prosthesis includes compensation for gaze location (Head and Eye, or Full Gaze), with congruent stimulation resulting in a perception that is spatially stable. The fourth column depicts what is perceived by the subject where the prosthesis delivers the image from the uncompensated camera (as in head-steered devices), but the brain continues to apply its knowledge about eye position, creating spatially incongruent conditions whenever the camera and eyes are not pointing in the same direction.

Visual prostheses create images by stimulating patterns of phosphenes. Eye movements that could be used to drive spatial updating in visual prostheses cause phosphenes to shift with each new position, just as eye movements in normal vision cause images on the retina to shift. This observation has a cause with easily-overlooked consequences: phosphenes are retinotopically locked, that is, they appear in a fixed position relative to the current gaze location. Brindley and Lewin recognized this effect in their seminal 1968 report on cortical stimulation^36^, as was confirmed by later studies^5, 37–45^. In normal vision, each time the eye moves to a new position, a different part of the visual scene falls on the retina and the brain uses its knowledge of gaze position to compensate the new input to correctly assemble a stable perception of the external world. An important limitation of head-steered prosthetic devices is that they do not update visual information as patients move their eyes to scan the environment, but the brain still is aware of eye position, creating a conflict (Fig. 1). By using cameras that are fixed in position relative to the head, typically on a set of goggles, such systems fail to maintain alignment of the camera and pupillary axes as the eyes move, and require implanted patients to direct their field of view solely with head scanning, while suppressing eye movements^14, 15^, an unnatural behavior that requires substantial training^46, 47^. Misalignments between the camera position and the patients’ gaze position impair perception and decrease performance in simple object localization tasks^48^; even minor misalignments between the eye and camera optical axes are known to affect performance in prosthesis user^49^.

Presenting images in eye-centric coordinates through a *gaze contingent* mechanism would potentially increase the usability of these devices^50^. Ideally, the imaging system (e.g., a goggles-mounted camera) should reproduce the view based on instantaneous eye position as if the camera had moved like the eye. Perhaps the most direct method to achieve this compensation would be to electronically shift a region of interest from a wide field of view so as to track eye-driven changes in gaze position^5^.

Recent attempts to introduce gaze contingency in simulation studies of artificial vision have opted for the incorporation of an eye tracker into the simulated prosthetic device^5, 40, 51–55^. Simulation studies with sighted individuals have found improved performance in gaze contingent conditions as compared to uncompensated ones^52, 54^. McIntosh^52^ employed a simulated artificial vision paradigm to simulate a retinal prosthesis with residual natural vision where sighted participants performed three visually-guided tasks, a search task to locate an object among distractors, a reach-and-grasp task, and a navigational task. For the search task, the time to locate the target object was reduced by up to a factor of 2 for gaze-contingent as compared to head-steered conditions. For the reach-and-grasp task, the improvement in completion time was up to 50%, with up to five times fewer touches on erroneous targets. For the navigational task, walking speed was improved by 10% and obstacle bumps reduced by up to 40%. More recently, Titchener and colleagues^54^ examined the effect of gaze eccentricity on hand–eye coordination by employing a simulated artificial visual paradigm, where the phosphene layout was modeled after Bionic Vision Australia’s second-generation retinal implant. Sighted participants performed a target localization task, where subjects reported the observed location of large (5° diameter) targets with their finger on the touch-sensitive screen. The gaze-compensation condition led to a significant reduction of pointing error when compared with the head-steered condition in six out of seven subjects. Importantly, in the head-steered condition, larger gaze eccentricity led to larger pointing error that was biased in the direction of gaze location, an effect that did not appear in the gaze-compensated condition.

In line with simulation studies with sighted volunteers, the effects of gaze-contingent versus head-steered stimulation have been described in studies with Argus II users^15, 48^. In a study by Sabbah and colleagues^48^, patients performed a target localization task where they were instructed to shift their gaze toward different locations on a screen by moving their eyes alone and maintaining their head still before pointing at the target. Rather than pointing at the actual target locations, subjects indicated positions on the touch screen that were biased in the direction the eyes were pointing. As there was no concurrent change in head position, and therefore no change in camera output, their result indicates that camera direction alone was insufficient for correct spatial localization when eye movements caused a misalignment between gaze and camera directions. In a study by Caspi and colleagues^15^, Argus II users were fitted with an experimental eye tracker which was enabled or disabled on a similar target localization touch task. When gaze contingency was enabled, head motions were significantly reduced and often nearly eliminated with mean head speed reduced by over half, and the extent of eye movement increased by a mean of 70%. While such changes might be expected with the introduction of an additional degree of freedom, importantly, no training was provided for the new mode, and pointing accuracy on the task improved by an average of 30% (see Discussion).

Research conducted so far to directly answer questions of the importance of gaze compensation suggests that it is indeed a critical aspect of visual prosthetics. Thus far, studies have either employed simple target detection tasks that do not capture the complexity of activities performed in everyday life^15, 48, 54^, or used relatively large and simple optotypes^56^, leaving open the question of the impact of gaze compensation in more cognitively engaging, naturalistic tasks.

Here, we address this gap by exploring the effect of gaze contingency with a reading task. Specifically, we created a virtual reality simulation of prosthetic vision that could be switched between modes where the simulated head-mounted camera produced a video stream that was either (a) corrected for gaze position, a mode that we call *Full Gaze*, or (b) steered only by head position, a mode that we call *Head Only*. As a metric of system utility, we used a reading task derived from the MNREAD test of visual acuity^57^, and compared reading accuracy and speed under the two experimental conditions. In our simulations, the Full Gaze condition, with inclusion of gaze position compensation, resulted in a marked increase in reading performance over the Head Only condition. This finding has strong implications for future visual prosthesis designs in order to maximize performance in tasks of daily living.

## Results

Subjects (*n* = 23, 9 male and 14 female, mean age of 26 years old, range 18–44 years old) were recruited from students at the University of Athens and the general population around the university and required to have proficiency in English (see Methods). They were verified to have normal vision with an informal Snellen chart evaluation, with binocular results of 20/13, or − 0.2 ± 0.1 logMAR (mean, s.d.; range of − 0.3 to 0.0 logMAR). Importantly, the assessed visual acuities were far better than what was required for the experimental task.

Subjects were presented a series of simple, three-line sentences to read on a computer monitor that simulated artificial vision from a device with 2000 phosphenes under the Full Gaze and Head Only conditions. The sentences were taken from the MNREAD corpus^57, 58^ and used to develop performance curves versus font size through the measurement of reading accuracy (percentage of words read correctly) and reading speed (number of correctly read words per minute). *Normal* mode trials without the artificial vision simulation were included as a control condition. The experiment was broken into a series of six-trial mini-blocks where each mini-block presented text for a given viewing condition at the six font sizes (see Discussion). Mini-blocks were ordered so that Normal (A), Full Gaze (B), and Head Only (C) conditions were each presented twice (A_1_, B_1_, C_1_, B_2_, C_2_, A_2_). Full details are provided in the Methods section.

### Reading accuracy and speed

**Figure 2:**
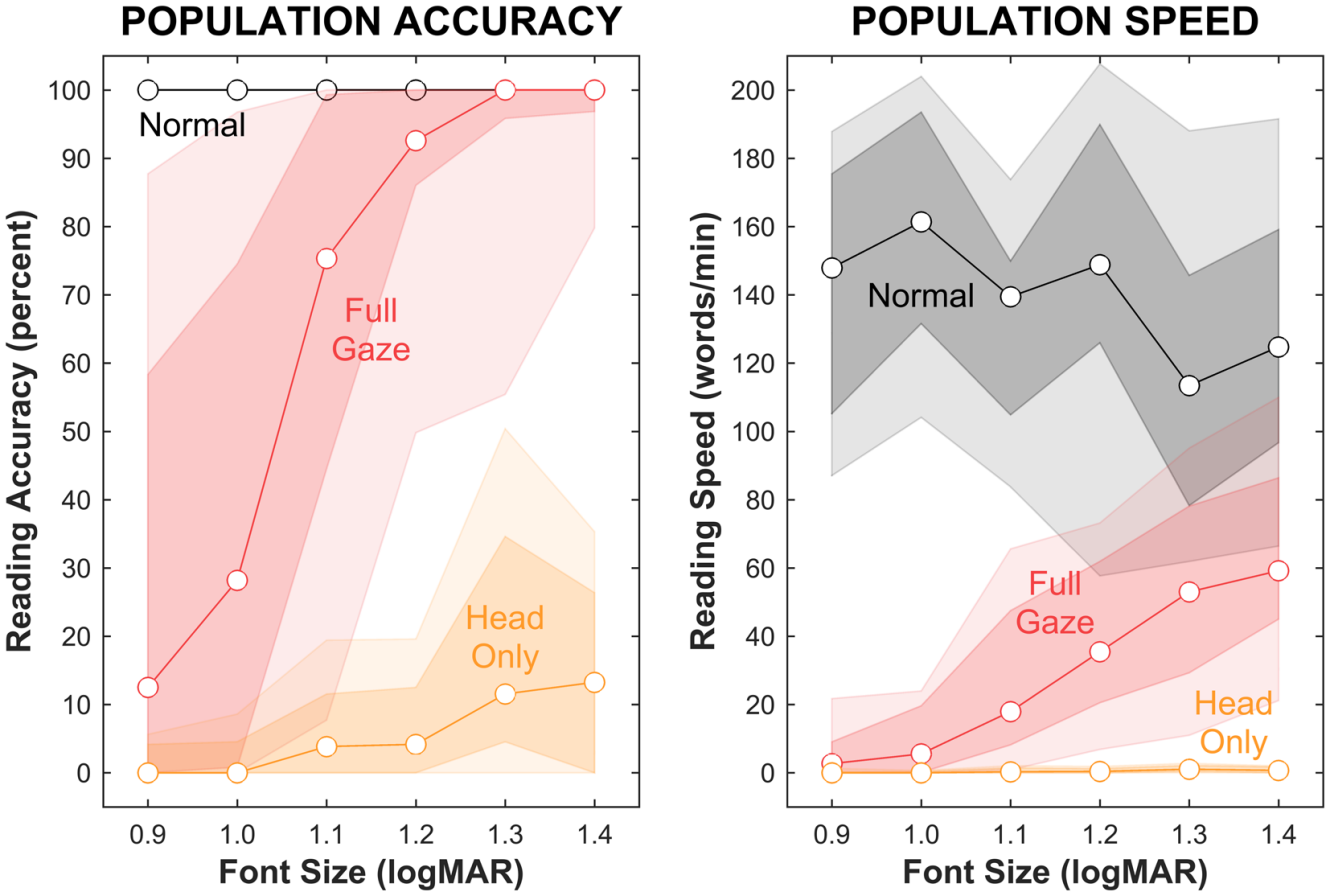
Population reading accuracy and speed. Reading accuracy (left) and speed (right) are shown for the three viewing conditions with population median as lines, 16/84 percentile range as dark color, 5/95 percentile range as light color. Performance is shown across the range of font sizes and measured in percent of correctly read words for accuracy, and number of correctly read words per minute for speed. The three conditions are Normal, where text is shown unadulterated on the screen (blacks), Full Gaze, where text is shown in a simulation of phosphene vision with full gaze compensation of the scene camera (reds), and Head Only, where text is shown in phosphene vision where the scene camera is steered only by head motions (oranges). In both phosphene view cases, the phosphene locations are locked to retinotopic location based on the instantaneous measured gaze position; we expect phosphenes for all visual prostheses to be stable in the retinotopic coordinate system (see Introduction). In the Full Gaze condition, the simulated visual prosthesis is assumed to incorporate a gaze tracker that provides rapid compensation for eye movements, performing a translation of the scene camera image as if it were being steered by the eyes. In the Head Only condition, gaze compensation of the scene camera is disabled, rendering it steerable only through head motion; this reflects the operation of many contemporary visual prosthesis designs.

Our primary finding is that both reading accuracy and speed were substantially higher for the Full Gaze condition than for Head Only for the font sizes measured (Fig. 2; Table 1). Performances under the three conditions are distinct by inspection for both metrics (Wilcoxon rank sum tests between Full Gaze and Head Only conditions pooled over all font sizes produce *p* = 10^–31^ and 10^–35^, *z* = 11 and 12, *r* = 1.0 and 1.1, on accuracy and speed respectively; individual font-size-by-font-size comparisons produce similar results with a maximum of *p* < 0.001, some of which are detailed below).

**Table 1:** Reading accuracy and speed results.

Measurement	Viewing condition	logMAR					
		0.9	1.0	1.1	1.2	1.3	1.4
**Reading accuracy (percent)**	Normal	$100.0_{ - 0}^{ + 0}$	$100.0_{ - 0}^{ + 0}$	$100.0_{ - 0}^{ + 0}$	$100.0_{ - 0}^{ + 0}$	$100.0_{ - 0}^{ + 0}$	$100.0_{ - 0}^{ + 0}$
	Full gaze	$13_{ - 13}^{ + 46}$	$28_{ - 27}^{ + 46}$	$75_{ - 31}^{ + 25}$	$96_{ - 7}^{ + 7}$	$100_{ - 4}^{ + 0}$	$100_{ - 3}^{ + 0}$
	Head only	$0.0_{ - 0.0}^{ + 4.2}$	$0.0_{ - 0.0}^{ + 4.5}$	$3.8_{ - 3.8}^{ + 7.7}$	$4.2_{ - 4.2}^{ + 8.3}$	$12_{ - 7}^{ + 23}$	$13_{ - 13}^{ + 13}$
**Reading speed (WPM)**	Normal	$148_{ - 43}^{ + 28}$	$161_{ - 30}^{ + 32}$	$140_{ - 25}^{ + 10}$	$149_{ - 23}^{ + 41}$	$113_{ - 35}^{ + 32}$	$124_{ - 28}^{ + 34}$
	Full gaze	$2.7_{ - 2.7}^{ + 6.4}$	$5.5_{ - 5.4}^{ + 14.1}$	$18_{ - 10}^{ + 30}$	$35_{ - 15}^{ + 27}$	$53_{ - 24}^{ + 25}$	$60_{ - 14}^{ + 27}$
	Head only	$0.0_{ - 0.0}^{ + 0.3}$	$0.0_{ - 0.0}^{ + 0.4}$	$0.3_{ - 0.3}^{ + 1.1}$	$0.4_{ - 0.4}^{ + 0.8}$	$1.0_{ - 0.6}^{ + 1.1}$	$0.7_{ - 0.7}^{ + 1.0}$

Values for reading accuracy in percent correct and reading speed in words per minute (WPM) are given for the three viewing conditions over the population of 23 subjects. Data are presented by font size and given as median values with differences to 16th and 84th percentiles. See Fig. 2 for a graphical presentation of these data.

For reading accuracy, under the Full Gaze condition, 19/23 (83%) subjects displayed 100% accuracy at the largest font size (1.4 logMAR); the population accuracy was $100_{ - 3}^{ + 0} \%$ (median and differences to 16th and 84th percentiles) with performance for the population tailing off over decreasing font size to $13_{ - 13}^{ + 46} \%$ at 0.9 logMAR, following a sigmoidal curve. In comparison, under the Head Only condition, subjects were only able to attain $13_{ - 13}^{ + 13} \%$ accuracy at the largest font size, tailing off to $0_{ - 0}^{ + 4} \%$ at the smallest size. For the largest font size, effect size between Full Gaze and Head Only conditions for Wilcoxon rank sum was *r* = 1.3 (with *z* = 6.0, *p* = 10^–9^), trailing off to 0.9 (*z* = 4.4, *p* = 10^–5^) at the smallest. The Normal condition of reading ordinary text confirmed there were no fundamental issues with $100_{ - 0}^{ + 0} \%$ reading accuracy at all font sizes. Sigmoidal fits of the population data for reading accuracy to estimate visual acuity at the 50% level showed the Full-Gaze condition provided an equivalent acuity of 1.02 ± 0.12 logMAR (95% confidence interval) and the Head-Only condition an equivalent acuity of 1.66 ± 0.37 logMAR (95% confidence interval; rank sum *p* = 10^–8^).

For reading speed, there was a larger contrast between the two phosphene view conditions than for reading accuracy. For the Full Gaze condition, speed at the largest font size was ${60}_{-14}^{+27}$ WPM, decreasing in a sigmoidal fashion to $2.7_{ - 2.7}^{ + 6.4}$ WPM at the smallest font size (Fig. 2; Table 1). The Head Only condition revealed a profound problem, with reading speed at the largest font size of $0.7_{ - 0.7}^{ + 1.0}$ WPM (one word about every 1 $\frac {1}{2}$ min, or two orders of magnitude slower than the Full Gaze condition) and with subjects barely able to read any words at the smallest font size ($0.0_{ - 0.0}^{ + 0.3}$ WPM). For the largest font size, effect size was *r* = 1.2 (*z* = 5.8, *p* = 10^–9^), trailing off to 0.9 at the smallest (*z* = 4.5, *p* = 10^–6^). Reading in the Normal condition that served as a control increased from $124_{ - 28}^{ + 34}$ WPM at the largest font size to $148_{ - 43}^{ + 28}$ WPM at the smallest (the smaller font sizes require shorter gaze shifts, so reading is somewhat faster), consistent with a previous report from a highly similar population^53^, and verifying that normal reading was possible.

**Figure 3:**
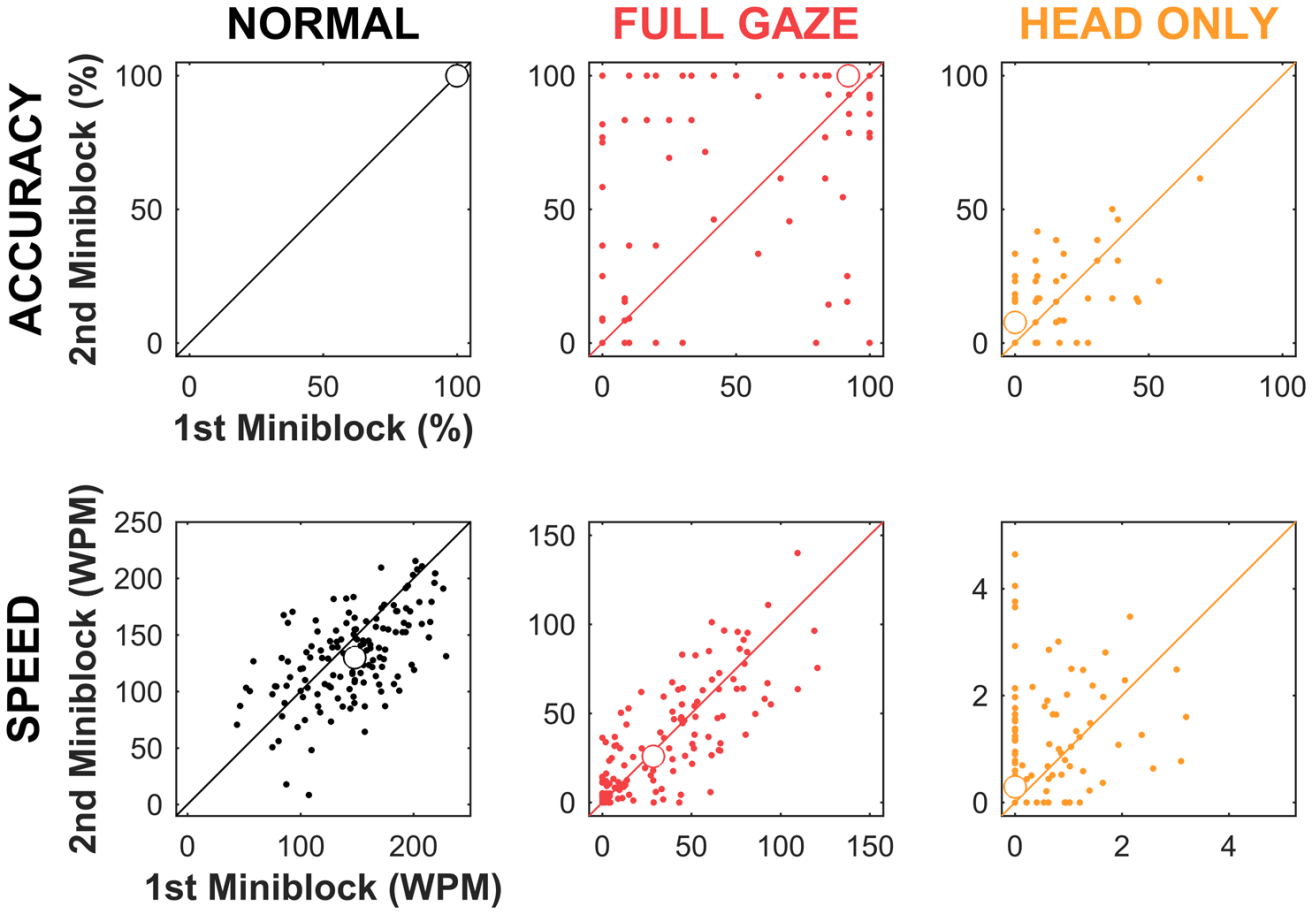
Performance in first versus second mini-block. Performance on reading accuracy (top row) and reading speed (bottom row) for the three viewing conditions, Normal (black), Full Gaze compensation (red), and Head Only steering (orange) is shown for the first versus second mini-block in each condition. All conditions had two mini-block presentations, each of which included one trial for all font sizes. Dots show the performance of matched stimulus conditions on the two mini-blocks. Unfilled circles show the population mean of the scattergrams. For the Normal condition, reading accuracy did not produce meaningful data as accuracies were all at 100%.

We examined performance between the first and second presentation of each class of mini-blocks to look for learning or other longitudinal effects (Fig. 3). For reading accuracy, there was no significance found for the Normal condition (Wilcoxon rank sum test, *p* = 1.0), with the median values being $100_{ - 0}^{ + 0}\%$ for both A_1_ and A_2_ mini-blocks across all font sizes. For the Full Gaze condition, there was, again no significance found (*p* = 0.2) with the median values being $92_{ - 84}^{ + 8} \%$ for B_1_, and $100_{ - 88}^{ + 0}\%$ for B_2_, suggestive of an improvement and consistent with what was found in an earlier report[53]. For the Head Only condition, a significant difference was found (*z* = 2.5, *r* = 0.2, *p* = 0.01) with median values of $0.0_{ - 0.0}^{ + 17}\%$ for C_1_ and $7.7_{ - 7.7}^{ + 13}\%$ for C_2_.

For reading speed, there was a significant but small (*z* = 2.8, *r* = 0.2, *p* = 0.005) slow-down in the Normal condition, from $148_{ - 48}^{ + 43}$ to $130_{ - 35}^{ + 41}$ WPM for A_1_ to A_2_. For the Full Gaze condition, there was no significance (*p* = 0.9) between B_1_ and B_2_ at $29_{ - 28}^{ + 38}$ and $26_{ - 38}^{ + 24}$ WPM, respectively. For the Head Only condition, there was a significant difference (*z* = 3.0, *r* = 0.3, *p* = 0.003) between C_1_ and C_2_ at $0.0_{ - 0.0}^{ + 1.0}$ and $0.3_{ - 0.3}^{ + 1.4}$ WPM, respectively. The C_1_ to C_2_ increase corresponds to one additional correctly read word (from zero to one, or from one to two) in the three largest font sizes. This small difference may reflect a leftward movement of the sigmoid curve of performance versus font size expected from a learning process^53^.

We compared average performance under the two experimental conditions on a per-subject basis to see if there were correlations (did being better at one viewing condition mean subjects were better at another?). No strong correlations were found. Comparing mean reading accuracy averaged over all font sizes for each subject, Spearman’s rank correlation was *r*_s_ = 0.36 with *p* = 0.09 between Full Gaze and Head Only conditions: good over-all performance on one condition had limited predictive power for the other. For mean reading speed, similar values were found at *r*_s_ = 0.37 with *p* = 0.09, again showing a lack of strong relationship. Lower levels of correlation that were also not significant were found for mean reading speed in the Normal condition against Full Gaze (*r*_s_ = 0.08, *p* = 0.7) and Head Only conditions (*r*_s_ = − 0.11, *p* = 0.6).

**Figure 4:**
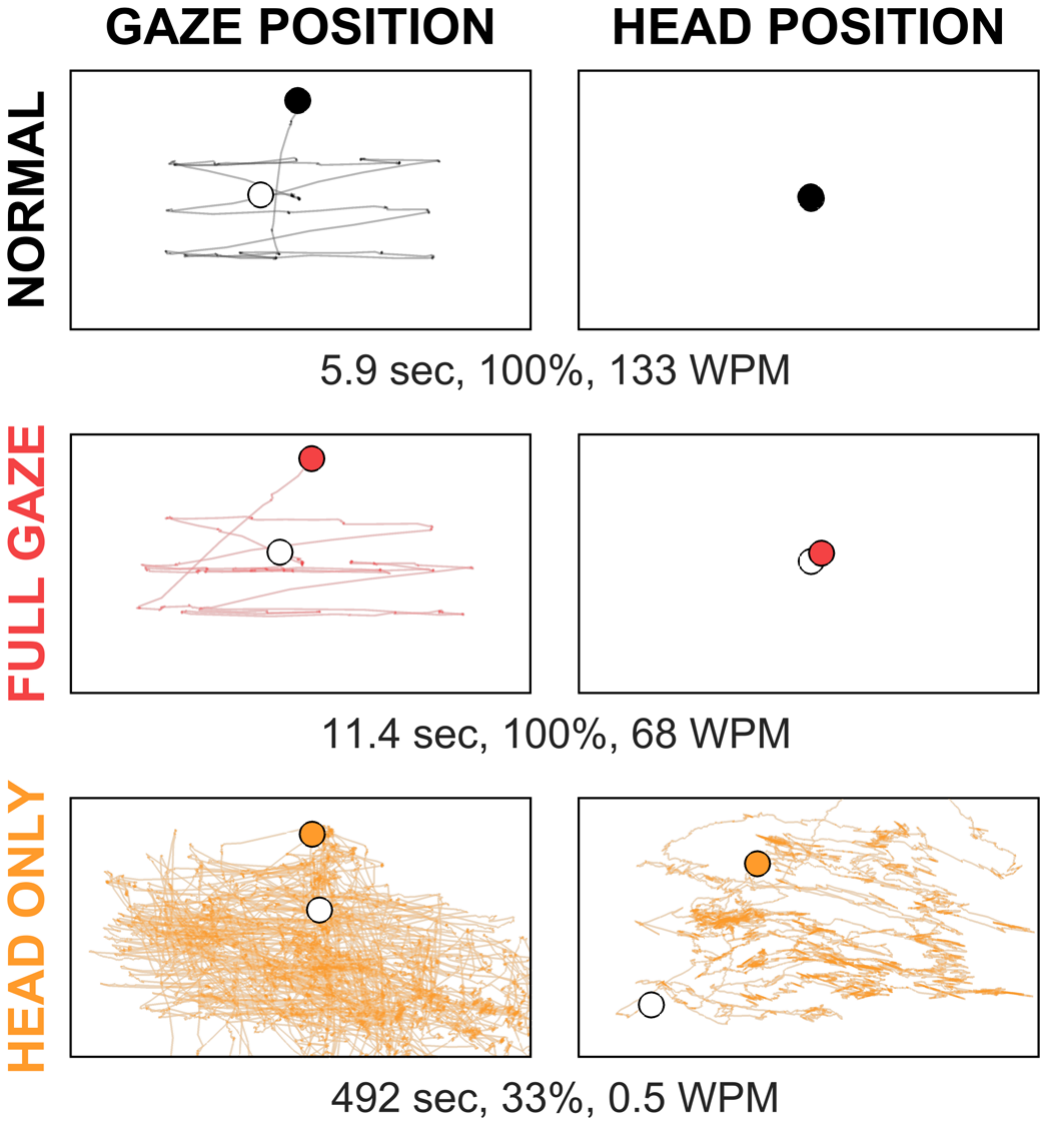
Example gaze and head traces from one subject. Gaze (left column) and head (right column) position traces are shown for one of the most overall-capable subjects reading text at 1.4 logMAR (the largest size) under the three viewing conditions, Normal (top row), Full Gaze (middle row), and Head Only (bottom row). For each trace, the start is shown by an unfilled circle, and the end by a filled circle. Trial length, reading accuracy, and reading speed are shown underneath each pair of plots: the first two trials (top two rows) took some few seconds to complete while the third trial (bottom row) took over 8 min; for Head Only, the values are substantially better than the population means, but a similar dichotomy in trial completion time was typical across the population. Eye movements during the Normal and Full Gaze conditions here follow a typical scan path for reading the three lines in the MNREAD sentences; head position is nearly motionless, also typical, without having instructed the subject to hold their head still. Eye movements in the Head Only condition reflect stereotypical looping center-out-and-back movements, while head position reflects attempts to scan the scene. This looping behavior is triggered by eye movements to foveate a portion of text the subject wishes to view, followed by a realization that the text tracks their gaze location (Fig. 9), and a subsequent saccade back to the center of the screen.

Subject behavior for gaze and head position in the control and two experimental conditions showed a high degree of similarity between Normal and Full Gaze conditions, with gaze location tracing out the three lines of text and head position relatively still, but no similarity with the Head Only condition that exhibited little-to-no structure in gaze location or head location despite higher levels of activity (Fig. 4). The total time spent reading with text at 1.4 logMAR was $6.1_{ - 1.3}^{ + 2.0}$ seconds in the Normal condition, $13.2_{ - 4.3}^{ + 3.9}$ seconds in the Full Gaze condition, reflective of a reading speed about half as fast (at the same 100% accuracy), and $138_{ - 106}^{ + 104}$ seconds in the Head Only condition, reflective of the substantially increased difficulty.

## Discussion

In this study, we assessed the importance of gaze contingency by employing a naturalistic reading task through a simulation of prosthetic vision that could be switched between two modes where the visual stimuli were either (a) corrected for eye position (*Full Gaze*), or (b) steered only by head position (*Head Only*). Performance was measured using the metrics of reading accuracy and reading speed that are well-studied in the low-vision literature, built upon the standard MNREAD test^57^, allowing the ready comparison and replication of our observations. Our primary finding is that under equivalent conditions, a simulated prosthesis that includes gaze compensation to determine how to activate phosphenes has substantial performance advantage over one that is limited to head steering of a camera. Although the differences in performance were most striking for the easiest conditions with large font sizes that activated many phosphenes, differences were still observable in the more difficult conditions with small font sizes that activated fewer phosphenes (e.g., the 1.0 logMAR text typically activated only 175 phosphenes for the straight-ahead gaze position). We suggest that our findings can be applicable to contemporary clinical head-steered devices with limited resolution and highlight the need to introduce gaze-contingent information to visual prosthetics by the incorporation of an eye-tracker to current head-steered devices.

### Reading performance is enhanced under full gaze compensation

Our findings show that reading performance in the Full Gaze condition can be relatively fluid and comparable to reading in the control condition, but becomes profoundly affected when participants were required to adopt a head-scanning strategy in the Head Only condition. This effect was evident in both accuracy and speed, as discussed in the following two subsections.

#### Reading accuracy

We obtained higher reading accuracy in the Full Gaze compared to the Head Only condition, replicating and extending previous work that has reported better performance under gaze-contingent versus non-gaze-contingent conditions^15, 52, 54^. A recent study by Caspi and colleagues^15^ had Argus II patients perform a pointing task to compare eye-and-head or head-only conditions analogous to our Full Gaze and Head Only conditions, respectively. They reported a pointing error of 3.7 ± 1.0 degrees with eye-and-head steering versus 5.1 ± 1.4 degrees with head-only (we compute *p* = 0.016 using a Wilcoxon signed rank test from their published data), for an immediate reduction in error of over 30% when engaging full gaze tracking with subjects who were already highly trained on head-only steering. Although that study and the present one are not directly comparable due to differences in the experimental design (localization versus reading task) and the subjects used in each study (blind implanted patients versus healthy, normal-sighted participants), our results are consistent with theirs, albeit our observations are that the effects are larger.

#### Reading speed

The differences between the Full Gaze and Head Only conditions were even more prominent for reading speed performance, which was deeply affected under the Head Only condition. This finding is in line with previous simulation work with sighted individuals showing faster completion times at a visual search task under a gaze compensated condition compared to a head-steered one^52^, as well as with reports that Argus II users adopt time-consuming compensatory head scanning strategies when presented with camera-gaze misalignments^48^. Our observation of the highest reading speed during Head Only condition being below 1 WPM (at 1.4 logMAR) is also consistent with reports from the literature for reading speed with actual visual prostheses that lack gaze contingency^56^. This consistency provides not only confirmation of our results, but by extension, an estimate for the level of performance increase that might be expected with the addition of gaze contingency to a clinical device. High-functioning Argus II users are reported to have single word reading accuracies of 71% with font sizes between 2.4 and 2.6 logMAR (see Table II in the 2013 report by da Cruz and colleagues^56^). Combining the results from that report with the range of completion times given elsewhere^59^, we estimate that these patients had a reading speed of between 0.2 and 0.9 WPM. While the experimental conditions were different from those presented here (larger font sizes used by da Cruz and colleagues^56^, single words versus full sentences, different phosphene counts and distribution), and their figures cannot be reliably translated to MNREAD measurements, the similar, low range of reading speed suggests that adding gaze contingency to the Argus II device may substantially improve its utility. While various factors might prevent the gain of two orders of magnitude in reading speed we reported above (for example, MNREAD sentences at 2.4 logMAR do not fit in a normal full visual field), it would not seem unreasonable to expect reading speeds in the range of multiple words per minute.

### Performance under the uncompensated Head Only condition can be improved

Although our task was not designed to test for possible learning effects, we observed performance enhancements between C_1_ and C_2_, the two Head Only mini-blocks. Learning effects have been already observed in gaze-contingent simulations of artificial vision^53, 60^, suggesting that training could create similar improvements in patients even with uncompensated devices. Given the case report of an individual who lacks the ability to move their eyes having spontaneously developed head-steering^61^, a natural follow-on question would be to see if training would benefit one mode more than the other, since we did not find a significant improvement for B_1_ to B_2_, the two Full Gaze mini-blocks. Discouragingly, training-driven improvements for camera-gaze misalignments have been found to be small and to progress at a slow rate^62^. We, thus, speculate that trained reading performance under the Head Only condition might not reach the levels we observed for the Full Gaze condition (see Post-implant rehabilitation and training, below).

### No training required for full gaze, invasive coaching required for head only condition

In addition to the striking differences in performance, we found that subjects did not require introduction or coaching to perform the first mini-block with phosphene view, B_1_, which employed the Full Gaze condition and resulted in naturalistic gaze shifts (Fig. 3). Subjects adapted to it quickly within the first trial and performed well, as expected from earlier work^53, 55, 60^. On the other hand, the Head Only mini-block C_1_ that followed required frequent coaching to, “keep your eyes looking forward and use your head to scan.” Without such coaching, subjects were, in general, unable to perform the task. The difference was not only striking, it was sobering for its implications for actual implants, as it touched the critical shortcomings that underlie approaches that do not support gaze steering, either intrinsically or through a compensatory mechanism. We are driven to the conclusion that the reports of seemingly poor performance in the literature that are in conflict with our previous work both in simulations with sighted humans^51^ and non-human primates^63^, and with implants in non-human primates in ongoing work are the results of the disadvantage faced by systems that lack gaze compensation. We further speculate that even with subject training to suppress gaze movements and employ head scanning, it will be difficult for prostheses lacking gaze compensation to match the utility of those that have incorporated it. Indeed, even if we were to further suppose that parity might be obtainable with the two approaches should non-gaze-contingent recipients undergo post-implant rehabilitation training, we have seen that an equivalent training effort with both simulated^53, 60^ and real gaze-contingent prostheses^64^, would result in substantial improvement in utility that would once again propel gaze-contingent systems to advantage. It is difficult to express how compelling the difference is between the two systems for naive subjects from our experiment: one is natural and fluid, and the other appears to require extensive training to establish even minimal utility.

With informal reports from our subjects that the Head Only condition induces mild nausea and vertigo, we can further speculate that one factor causing the relatively low retention rate of retinal prostheses in implanted patients^65, 66^ may be a lack of gaze contingency for certain devices. According to a recent report, two thirds of the Argus II patients used the device less than 5 h per week^66^. Fortunately, it is straightforward to augment nearly every current visual prosthesis design that lacks gaze contingency with a gaze tracker and upgraded external image processing to improve the experience for recipients, such as done on an experimental basis for the Argus II^15^. Taken together, based on our results and our subjects’ informal reports, we strongly encourage all visual prosthesis designs that currently lack gaze contingency to incorporate the feature as quickly as is practical.

### Post-implant rehabilitation and training

We expect training to be an important part of post-implant rehabilitation for foreseeable future prostheses as artificial vision is widely understood to be a crude approximation to normal vision, even for the most advanced, naturalistic designs. Our previous study on how reading ability changed with experience for a simulated thalamic visual prosthesis^53^ showed that active training provided substantial benefit, even in the case of phosphene counts not far from what are currently in clinical use. Although beyond the scope of this study, training effects would certainly be expected in the Head-Only condition with additional experience. We provide a speculative prediction immediately below.

Based on the amount of improvement seen in earlier work from our laboratory^53^, we can estimate the amount of improvement that would be expected here after extensive training. For the Full Gaze condition here, we have a close match to the conditions in the previous study—both included the same 2000-phosphene pattern, the same gaze tracker, the same gaze compensation algorithm, and observed highly similar initial performance—and thus we would expect reading accuracy averaged over the font sizes used here to reach 100% and equivalent acuity to improve from 1.0 to 0.7 logMAR in 10–15 sessions. For the Head Only condition, the closest match performance-wise is the lowest resolution pattern in that previous work, P_500_/Low. Understanding that the correspondence between the two conditions is poor (beyond one being gaze contingent and the other head steered), we can speculate that the Head Only acuity here might improve from 1.6 to 1.3 logMAR after 40 sessions and the mean reading accuracy might rise from 8 to 50%, based on the improvement in P_500_/Low acuity there. Under this highly speculative model, while performance under the Head Only condition here would improve with extensive training, it still would not match performance in the Full Gaze condition observed with naive subjects.

A recently published report^46^ of interviews of patients undergoing the training necessary for the Argus II device, which lacks gaze contingency, suggests that it is a difficult and unpleasant experience. An earlier case report^47^ describes similar training frustrations in one patient. Our results point to the lack of gaze contingency being a potentially important factor in these observations.

### Phosphene pattern, field extent, and theoretical acuity

The phosphene pattern used in our simulations (Fig. 8) is generated by an electrode array that is like many from current clinical prostheses in that it has a constant density in the tissue where electrodes are implanted. For constant tissue density of electrodes, the spacing of phosphenes in the visual field varies from area to area: in the retina, it results in an even spacing of phosphenes, but in LGN and visual cortex, it results in a pattern that includes effects of tissue magnification to create a distribution that is center-weighted and representative of the endogenous profile of acuity versus eccentricity^5, 67^.

Contemporary visual prostheses typically stimulate a limited extent of the visual field, but here, we have simulated a device that has electrodes that span the entirety of LGN, and thus creates phosphenes spanning the entirety of the visual field (Fig. 8). In this way, our simulation is intended to be forward-looking, rather than representative of devices in the clinic, and will not reflect the experience of a current prosthesis recipient, except insofar as to predict that the addition of gaze compensation to head-steered prostheses will result in an improvement in patient experience and device utility. Combining observations that have been made by others in such devices^15, 52, 54^ and the results presented here, we believe this prediction to be well-founded.

The pattern used here, with 2000 phosphenes, has a theoretical acuity of 1.1 logMAR within the foveal area (the central two degrees) that falls off with eccentricity and is 2.7 logMAR at a point corresponding to the left and right edges of the monitor in our simulation when the subject’s gaze is at the center. The central acuity in our pattern was therefore higher than the best reported values for Alpha AMS and IMS patients of 1.4 logMAR^68^, and for Argus II patients of 1.6 logMAR^69^, although of the two, only the latter device lacks gaze contingency.

### Linguistic ability was not a factor

While the subjects were non-native English speakers, they were required to present certification of college-level ability, and the MNREAD sentences use a 3^rd^ grade elementary school vocabulary. The observed high reading speeds and 100% accuracy in the Normal condition demonstrate that linguistic issues were not a limiting factor in our results.

### Eye tracking and calibration in blind individuals

The inclusion of an eye tracking device in a visual prosthesis design that does not inherently include gaze compensation brings the question of tracker calibration. Normally, calibration of eye tracking devices is done in sighted subjects by activating targets at known locations, an option that is not generally available with blind patients. Nevertheless, there are advanced approaches that allow basic calibration using only external measurements of the eye that do not depend on subject perception ^70–72^. We expect these approaches combined with a refinement thorough a perceptual process such as the pointing paradigms extensively described in the literature^15, 36, 45, 73, 74^, or staircase saccades, vestibulo-ocular reflex (VOR), smooth pursuit, or other continuous observation methods (see review by Kar and Corcoran^75^) will prove sufficient to reap significant benefits from incorporating gaze-contingency into visual prosthesis devices. While there is some concern about abnormal gaze shifts in blind patients, Alpha IMS implant recipients have been observed to have relatively normal eye movements, suggesting a re-provisioning of visual input has a normalizing effect^32^.

High quality gaze tracking was critical to support the accuracy of our simulations, but it will also play a role in implementing gaze contingency in prosthetic devices that would otherwise be head-steered. Current commercial goggles-mounted eye trackers (e.g., Tobii Pro Glasses 3, Tobii, AB; Pupil Core, Pupil Labs, GmbH) are approaching the accuracies of the hardware used in this work, suggesting that designers of future gaze-compensated prostheses will have capable tools at their disposal.

## Conclusion

Visual prosthesis devices that deliver a replacement for the visual information that is normally provided by the retina are constrained by the design of the visual pathway. The brain combines retinotopic information with information about the location of the eyes and the head to integrate stimuli from different gaze locations into a unified representation of the environment. As retinotopic information is perforce locked to gaze direction, artificial visual devices should optimally compensate their regions of interest to track instantaneous gaze position. We have demonstrated that in a simulation of artificial vision, including real-time gaze compensation makes the simulation easy to use, and reducing the simulation to a head-steered device that lacks awareness of eye position reduces the utility to near zero for naive subjects in a reading task. We conclude that adding eye tracking to existing devices should immediately improve usability and may impact patient retention.

## Materials and methods

### Participants

Twenty-three subjects took part in the study. The sample size was selected based on previous simulation studies from our laboratory with sighted individuals^51, 55^. Subjects were required to have self-reported normal or corrected-to-normal vision, no history of psychiatric or neurological impairment, and be able to read English text out loud. One participant was a native English speaker, while the rest (*n* = 22) were native Greek speakers with sufficient English language skills for the modest requirements of the task (Level B2 according to the Common European Framework of Reference for Languages). One additional subject (a 24th, data from whom are not otherwise reported), was disqualified during the consenting process due to strabismus. Subjects were assigned pseudonyms for the purpose of anonymizing data collection and they either volunteered or received course credits for their participation in the study.

### Ethics statement

The research in this report was performed according to a protocol approved by the Ethics Committee of the Department of History and Philosophy of Science at the University of Athens and the Institute Review Board at Massachusetts General Hospital and in compliance with the guidelines of the Declaration of Helsinki. This study was determined to contain minimal risk and informed consent was provided by each participant.

### Apparatus

**Figure 5:**
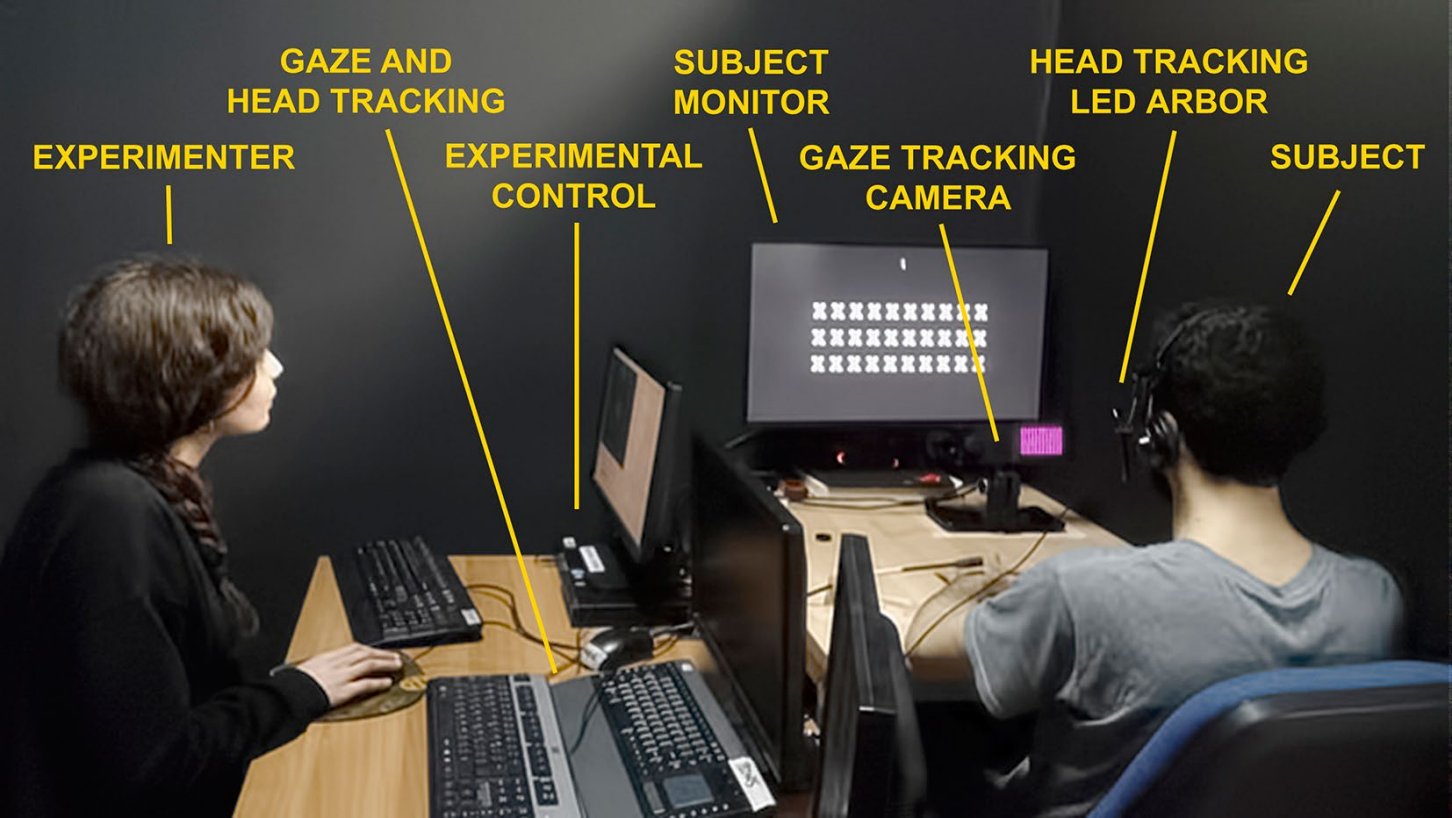
Apparatus. An apparatus was constructed to drive the simulation under the various viewing conditions. Subjects are seated at a table on which rests a high-refresh rate LCD monitor (ASUS, PG279Q, running at 100 Hz refresh), a gaze tracking camera (SR Research, EyeLink 1000 +), a head position tracking camera (TrackHat, opentrack), and a microphone. As part of the head tracker, subjects wear a set of headphones that are used only to hold a three-LED arbor so that it faces the head-tracking camera (Natural Point, TrackClip Pro). Simple sentences are presented on the subject screen in a simulation of artificial vision using phosphenes that are stabilized on the subject's retina based on the instantaneous report from the gaze tracker. A virtual scene camera is steered either through gaze location (Full Gaze condition) or head position (Head Only condition) in the simulation. As the subjects read the sentences aloud, scores are kept by the experimenter on sheets that are not visible to the subjects. The vision simulation and experimental control are run from a single computer (Lenovo Tiny M710p).

The system used in this experiment has substantial commonality with one that has been described in a previous publication^53^ using techniques that have been described in detail^55^. Subjects were seated in front of an apparatus that included a heads-free gaze tracker (EyeLink 1000 +, SR Research, Inc.), a head tracking system (TrackClip Pro, Natural Point, Inc., as infrared target; *opentrack* software, TrackHat, Ltd., to perform tracking; PS3eye, Sony, Inc., as imaging camera), and associated computers for processing gaze and head position, along with stimulus generation and experiment control (Fig. 5).

Visual stimuli were presented to the subjects on an LCD computer monitor (PG279Q, Asus, Inc.) operating at 1600 × 900 resolution and 100 Hz video refresh, placed 70 cm away from the subject. At that distance, the 59.9 cm by 33.6 cm extent of the monitor’s display subtended 41 by 24 degrees in the subject’s visual field. As described below, during the presentation of simulated phosphene vision, the display was updated on a frame-by-frame basis.

System latency was not directly measured, but is estimated to have been under 20 ms. The eye tracker was operating in 1000 Hz mode with 2-point averaging, for an effective 2 ms latency. The gaze position was sampled at the start of each 10 ms video refresh cycle and used to create a stimulus frame that was sent to the video monitor immediately prior to the next refresh for a total of 12 ms signal latency. The monitor itself was measured to have an additional 6 ms latency (Video Signal Input Lag Tester, Leo Bodnar Electronics, Ltd), which has been reported to be primarily pixel response time (TFT Central, http://www.tftcentral.co.uk/reviews/asus_rog_swift_pg279q.htm). Adding these three sources of latency together (2 + 10 + 6), gives us an estimate that we conservatively rate at 20 ms.

### Design and stimuli

#### Experimental conditions: primary experimental variable

**Figure 6:**
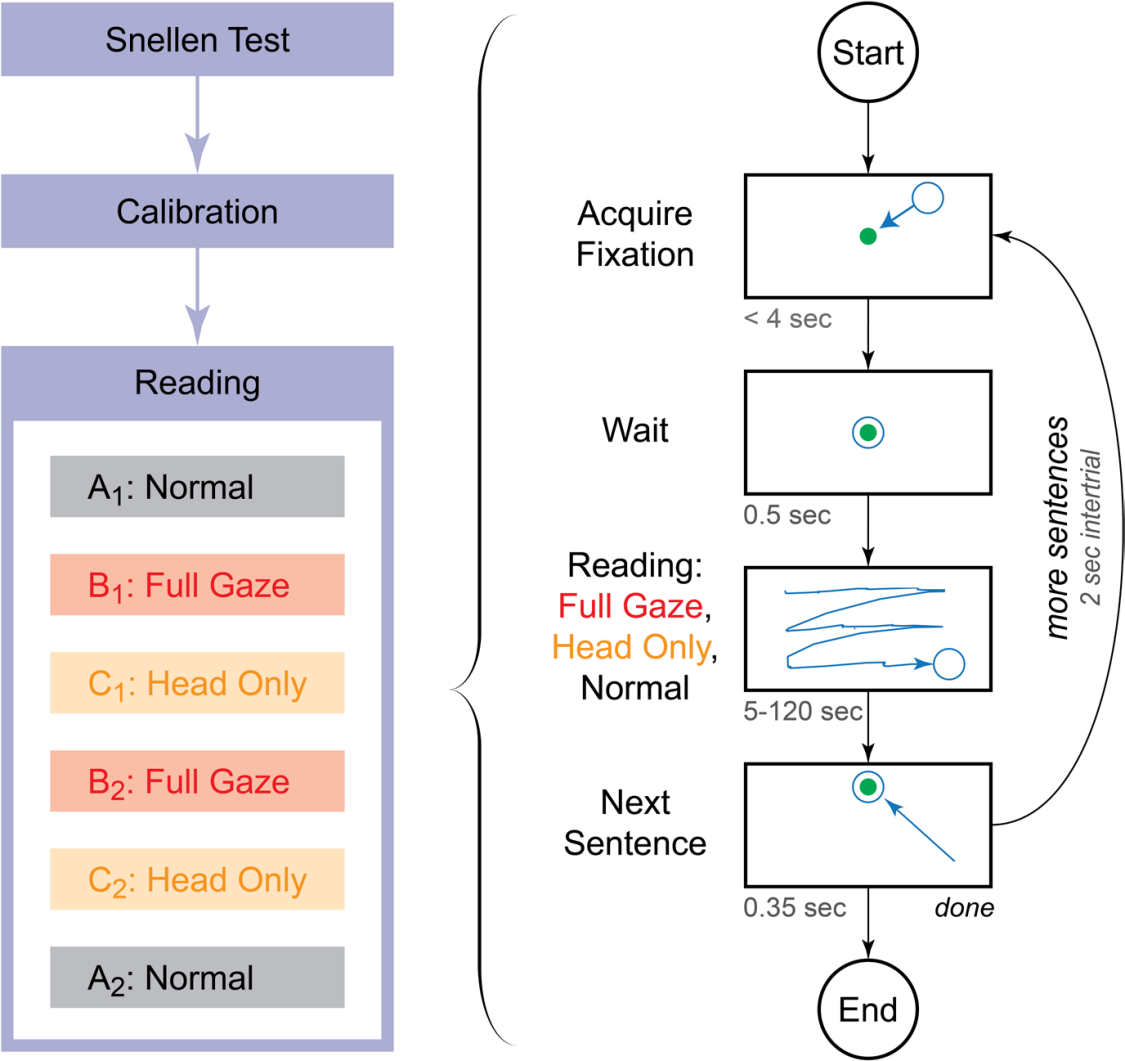
Overview of experimental design and procedure. (Left) Each subject’s session started with a coarse assessment of visual acuity using a traditional Snellen chart. This test was to verify that subjects had largely normal vision, rather than to precisely measure their visual acuity, as the experimental requirements were well below ordinary acuity. Then, after subjects were seated in front of the apparatus, a quick calibration of the gaze position system was performed, typically lasting 2–3 min. Finally, the experiment itself proceeded with the presentation of the six mini-blocks in sequence, starting with a control condition mini-block, followed by four experimental mini-blocks, and then closing with a second control mini-block. (Right) The reading task used a trial-based structure where each trial began with the subject fixating a central point using normal vision. After a brief pause, a simple, three-line sentence was presented on the screen in one of the three conditions, depending on the mini-block: the Normal condition using natural vision, and the Full Gaze and Head Only conditions using phosphene-view simulation to present the stimulus. In both phosphene view conditions, the phosphenes were stabilized on the retina; in the Full Gaze condition, the simulated scene camera was steered by the combination of eye and head positions, whereas in the Head Only condition, the scene camera was steered only by head position. For all three conditions, the subjects read the sentence aloud as best they could, and then fixated the Next Sentence dot to proceed to the next trial.

To investigate the effects of gaze contingency on reading performance in a simulation of artificial vision, we manipulated the gaze compensation mode. Specifically, we introduced three viewing conditions that were presented in mini-blocks (Fig. 6): (A) a *Normal* condition, where clear text was presented on the screen as a reference for normal reading behavior, (B) a *Full Gaze* artificial vision condition, where the simulated phosphenes were updated based on the position and direction of both the head and the eye, thus stabilizing the phosphene locations on the retina, and (C) a *Head Only* condition, where phosphenes were updated based on the position and direction of the head only. The conditions were presented in a mini-block fashion, as pilot data showed that participants encountered major difficulties in switching between Full Gaze and Head Only conditions on a trial-to-trial basis.

#### Letter size and text sentences: secondary experimental variables

**Figure 7:**
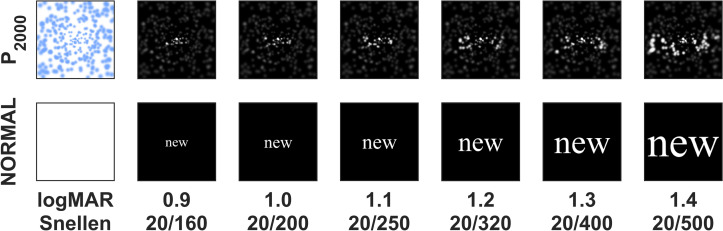
Stimulus conditions and font sizes. Example renderings of text used during the simulations is shown for the Normal condition (bottom row) where text is rendered at the native resolution of the screen, and the phosphene view conditions (top row) where text is shown as seen through a collection of 2000 phosphenes. The central part of the screen, approximately 10 degrees of visual angle across, is depicted for the six different font sizes used. Font sizes were calibrated to correspond to 0.9 logMAR (smallest) through 1.4 logMAR (largest). The central part of the phosphene pattern corresponding to the screen areas rendered here is shown in the upper left square, with each phosphene appearing as a light blue Gaussian on a white background. When this pattern is used as a filter on the white text/black background, it results in the images in the top row, for gaze and camera positions both at the center of the screen. As the gaze location moves around in either of the phosphene view conditions, the phosphene pattern is stabilized on the retina, but the scene camera is steered according to the testing condition of the mini-block (Full Gaze or Head Only).

Secondary experimental variables that were used to compute subject performance for each mini-block mode included font size of the text, carefully calibrated to be equivalent to 0.9 to 1.4 logMAR in steps of 0.1, and the text content of each sentence (Fig. 7). These secondary variables were used to generate psychometric curves and validate observations against previous reports^53, 55^.

Sentences were taken from the MNREAD corpus^57^ and presented in the Times New Roman font. Sentences in the MNREAD corpus have 60 characters, including spaces but not including a final period, include no punctuation, and no proper nouns. They are presented across three lines (Fig. 9), and have been extensively studied to ensure they have little variation in readability^53^.

#### Phosphene pattern

**Figure 8:**
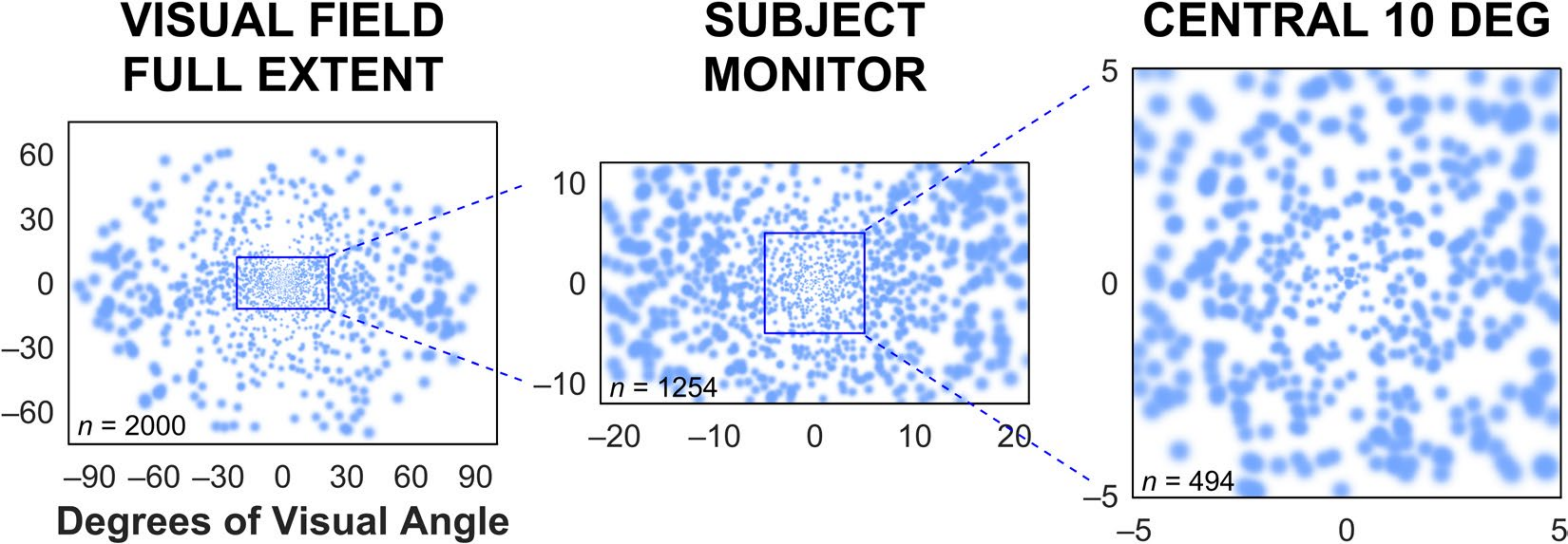
Phosphene pattern. The phosphene pattern used in this experiment contained 2000 phosphenes total, spread over the entire visual field in a center-weighted pattern that follows the natural profile of visual acuity from the center of gaze to the periphery^5, 67^, as seen in the left image. During the task, the phosphenes that fall on the subject monitor would be simulated. As the pattern is gaze-locked in retinotopic coordinates, the pattern is shifted with gaze movements during the simulation, and the number of phosphenes that fall on the monitor accordingly varies; that number would be maximum at about 1200 phosphenes when the gaze position was straight ahead, as seen in the middle image. In phosphene view mode, those phosphenes would be used as a filter on the image to be presented to the subject (see Figs. 7, 9). The central part of the visual field that carries the highest density of phosphenes is shown in the right image. For phosphene patterns in this class that model thalamic visual prostheses under development in our laboratory, the central 10 degrees typically has about one-quarter of the total phosphenes^51^.

As the intent was to simulate the style of devices under investigation in our laboratory, the pattern of phosphenes used were heavily center-weighted, but fully span the visual field (Fig. 8), as has been previously discussed^5, 67^. In particular, the phosphene pattern used here is based on a visual prosthesis design that places stimulating electrodes in the dorsal lateral geniculate nucleus (LGN). This thalamic approach affords advantages to retinal or cortical approaches that have been discussed in the literature ^4, 5, 23, 40, 51, 55, 63^, and include applicability to a wide range of disease conditions and causes of blindness. Importantly for the present work, the distribution of phosphenes in a given implant is expected to reflect the endogenous distribution of visual acuity across the visual field with resolution highest at the point of regard with small, densely packed phosphenes, falling away toward the periphery with large, sparse phosphenes (see Discussion).

### Procedure

The experimental procedure was divided into three parts: (1) Snellen acuity assessment, (2) subject seating and calibration of the gaze tracker, and (3) the main experiment which was subdivided into the six mini-blocks (Fig. 6). A full session with a given subject took about 30 min.

#### Snellen acuity task

Each participant was administered a standard Snellen chart task to verify that they had approximately normal vision. Subjects stood at a measured mark, 3 m away from a wall-mounted Snellen chart. Normal overhead office lights were used to generate ordinary levels of illumination. The test was administered binocularly with corrective lenses if the subject normally used them, and at a pace determined by the subject. Responses were converted to logMAR units.

#### Initial seating

After the Snellen screening, participants were brought to the experimental room, and seated comfortably in front of the subject monitor at a distance of about 70 cm from the display. Subjects wore both a bulls-eye sticker on their forehead to support frame-free gaze tracking, and a set of non-functional headphones onto which the head-tracking LED arbor was mounted. To improve the quality of tracking results, subjects were seated with their chest against the table and arms on the table to create a natural centering of head position without undue limits on head rotation. Instructions were given for the calibration task which was then performed, followed by instructions for the reading task which was then in turn performed.

#### Calibration task

Calibration of the gaze tracking system has been described previously^53^ and is summarized here. Participants were presented a series of small dots one at a time in an array of locations that spanned the screen and were instructed to fixate each as closely and as accurately as possible, maintaining fixation for the duration of each dot. The first thirteen presentations (one for each location) were used to trigger EyeLink 1000 + calibration while the subsequent 27 presentations (three instances for each of nine locations) were used to calibrate a secondary non-linear correction in the experimental software. The head position tracker did not require per-subject calibration as it used a fixed LED arbor of known geometry.

#### MNREAD-based reading task

The MNREAD task^57, 58^ assesses visual acuity by presenting a series of simple three-line sentences that are read aloud. Both reading speed and accuracy are tracked as the size of the font used to display the text is varied, typically resulting in logistic-like psychometrics where decreasing text size results in decreasing performance. The MNREAD task can be considered an alternative to single letter acuity tasks such as the familiar Snellen or Sloan charts that are common in clinical use. In the laboratory setting, we find MNREAD sentences are an efficient and effective way to collect acuity-related data with human subjects; the variant we have used here has been validated previously^55^.

The main experiment consisted of three mini-blocks of 6 trials each (Fig. 6, left panel). Each mini-block was presented twice and the order of presentation was kept constant across participants. Each participant completed 36 trials in total. We used a mini-block structure, rather than a fully interleaved method, due to the unanticipated difficulty of the Head Only condition discovered during development. Within each mini-block, each of the six font sizes was presented once in pseudo-random order that varied from mini-block to mini-block, but was conserved across subjects. The mini-block sequence presented viewing conditions in order of increasing difficulty (Natural Vision, Full-Gaze, Head-Only) so as to ensure any bias introduced by the sequence would diminish, rather than enhance, any differences from condition to condition.

**Figure 9:**
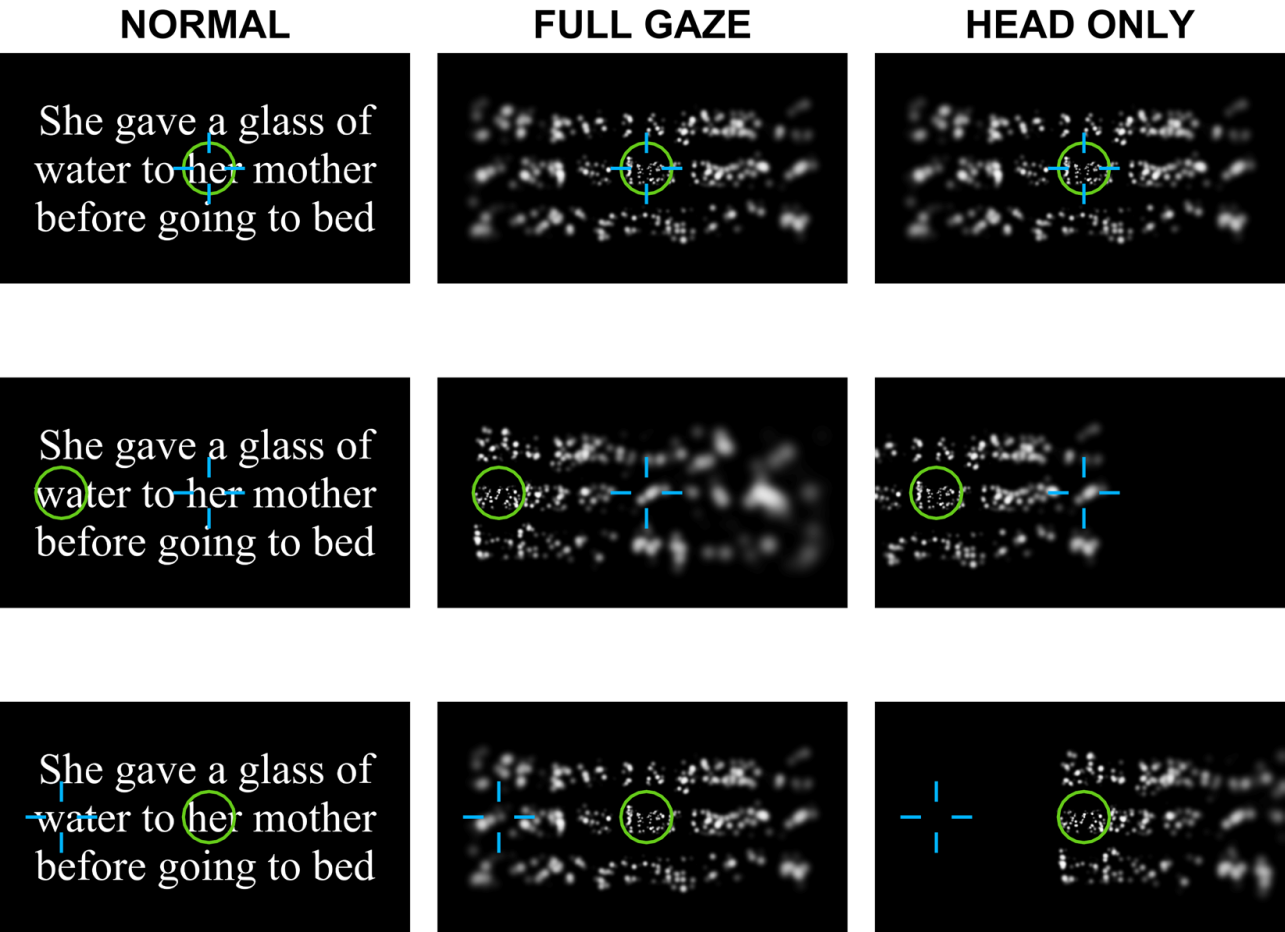
Screen captures from experimental conditions. Example screen shots are shown from the three conditions for an example sentence at the largest font size, 1.4 logMAR. The three columns correspond to the Normal (left column), Full Gaze (middle), and Head Only (right) conditions. The three rows correspond to three different alignment conditions for gaze (green circle) and head (blue cross) positions: the first (top row) contains the stimuli displayed under the three conditions when both the gaze and head positions are straight ahead, at the center of the screen where the subject is looking at the word /her/; the second (center) contains stimuli when the gaze is left (eyes rotated left), but the head is straight such that the subject’s gaze is on /water/, but the camera remains directed at /her/; the third (bottom) is for the gaze straight (eyes counter-rotated right) and head left such that the gaze is again on /her/, but the camera is on /water/. When the eyes are deviated from straight forward in the head, the resulting disagreement between gaze and head position creates spatially incongruent stimuli for Head Only conditions that was found to be highly disconcerting to subjects and resulted in very poor performance compared to the Full Gaze conditions that are sensitive to eye position.

Each trial was subdivided into a series of four phases (Fig. 6, right panel), *Start*, *Pre-Stimulus*, *Reading*, and *End*. During the Start Phase, a fixation point appeared in the middle of the screen that the subject was required to foveate in order to engage the experiment. The color of the fixation point served as a visual cue informing participants about the viewing condition of the trial (white dot indicating the Normal condition; red dot, Full Gaze condition; orange dot, Head Only condition). Once foveated for the duration of the Pre-Stimulus Phase, the fixation point disappeared. After the fixation offset, the Reading Phase started with one of the sentences displayed with either no adulteration for the Normal condition (ordinary, plain text shown on the monitor), phosphene view with gaze-compensation for the Full Gaze condition, and phosphene view without sensitivity to eye position for the Head Only condition, each at one of the font sizes (Figs. 7, 9). An additional *Next Sentence* dot, which matched the color of the trial’s fixation point, was displayed near the top center of the screen. Subjects were instructed to read each sentence out loud as best as they could while skipping words they were unable to discern, or to state that they were unable to read the text at all. Subjects were instructed to look at the Next Sentence dot in order to advance to the next sentence. Subjects could take as long as they wanted, consistent with reading quickly and accurately. Once subjects foveated the Next Sentence dot for 350 ms, the trial entered the End Phase, the screen was blanked, and a 2000 ms pause provided an intertrial interval before the onset of next trial. Each trial used a different MNREAD sentence, but the sequence of sentences and conditions was maintained across subjects. An audio recording was made for the entirety of each experiment.

To ensure an accurate simulation and reading performance assessment, the text for each trial was rendered off-screen with compensation for the head-to-screen distance as measured during the pre-stimulus portion of the trial. An ideal simulation might re-render the reference image of the text with each frame, but with the hardware at hand, that would have unacceptably slowed the simulation.

However, there was sufficient computational power available to adjust the diameter of phosphenes as drawn on the screen to the per-frame viewing distance so that the sizes on the retina were approximately constant. This compensation was computationally inexpensive, and thus readily incorporated without impact on the simulation.

Accurate measurements of reading speed required an accurate assessment of the time spent in reading for each trial. During the developmental phase of the experiment it became clear that if viewing conditions were interleaved on a trial-by-trial basis, a substantial amount of time was being spent at the start of each trial for the subject to figure out the current experimental mode. For Full-Gaze trials, this probing did not add as much of an artifactual latency as for Head-Only trials; thus, to reduce bias, a mini-block structure was selected for collection of the primary data reported here.

### Data analysis

#### Reading accuracy

Each subject's orally provided responses were compared to a printed version of the MNREAD sentences in order to score the number of correctly read words for each sentence. Counts were then normalized by sentence length and multiplied by 100 to find the reading accuracy, or percentage of correctly read words for each combination of gaze compensation mode and font size. Each combination was presented twice during a session (once per mini-block), but with two different sentences, and the mean value over the two presentations was used for the subject's performance on that combination. Control comparisons were performed between the first and second mini-block for each condition to rule out longitudinal effects. An equivalent visual acuity was computed by measuring the 50% point along a logistic curve fitted to reading accuracy versus font size. As reading accuracy has a bounded range, median values with 16/84 percentile ranges are reported in lieu of means and standard deviations.

#### Reading speed

The reading speed was computed as the number of correctly read words per minute, for each combination, again averaged over the two presentations. The length of time for a given trial was measured from the time the sentence text appeared on the screen until the subject foveated the Next Trial dot. In Head Only trials, detection of Next Trial foveation was based on normal gaze position on the screen, rather than head-steered aiming, and was computed independently from stimulus presentation. As with Reading Accuracy, reported values are medians with 16/84 percentile ranges.

#### Expectations

For both reading accuracy and reading speed, we expected to observe significantly higher performance for the Full Gaze compared to the Head Only condition. Reading accuracy is not normally distributed because of its limited range, an effect that becomes especially apparent near 0% or 100%. Reading speed has a similar lack of normality for very low values like the ones we observed. To test our hypotheses, we determined statistical significance using Wilcoxon rank sum tests as the assumption of normality was not met using a Kolmogorov–Smirnov test for normality in many cases. For statistical comparisons, a *p* value below 0.05 was considered to be significant.

## References

[1] Chuang AT, Margo CE, Greenberg PB (2014). Retinal implants: a systematic review—Table 1. Br. J. Ophthalmol..

[2] Donaldson N, Brindley GS, Shepherd RK (2016). The historical foundations of bionics. Neurobionics: The Biomedical Engineering of Neural Prostheses.

[3] Goetz GA, Palanker DV (2016). Electronic approaches to restoration of sight. Rep. Prog. Phys..

[4] Mirochnik RM, Pezaris JS (2019). Contemporary approaches to visual prostheses. Mil. Med. Res..

[5] Pezaris JS, Eskandar EN (2009). Getting signals into the brain: visual prosthetics through thalamic microstimulation. Neurosurg. Focus.

[6] Schiller PH, Tehovnik EJ (2008). Visual prosthesis. Perception.

[7] Edwards TL, Cottriall CL, Xue K, Simunovic MP, Ramsden JD, Zrenner E, MacLaren RE (2018). Assessment of the electronic retinal implant Alpha AMS in restoring vision to blind patients with end-stage retinitis pigmentosa. Ophthalmology.

[8] Stingl K, Bartz-Schmidt KU, Besch D, Braun A, Bruckmann A, Gekeler F, Greppmaier U, Hipp S, Hörtdörfer G, Kernstock C, Koitschev A, Kusnyerik A, Sachs H, Schatz A, Stingl KT, Peters T, Wilhelm B, Zrenner E (2013). Artificial vision with wirelessly powered subretinal electronic implant Alpha-IMS. Proc. R. Soc. B Biol. Sci..

[9] Stingl K, Bartz-Schmidt KU, Besch D, Chee CK, Cottriall CL, Gekeler F, Groppe M, Jackson TL, MacLaren RE, Koitschev A, Kusnyerik A, Neffendorf J, Nemeth J, Naeem MAN, Peters T, Ramsden JD, Sachs H, Simpson A, Singh MS (2015). Subretinal visual implant Alpha IMS: clinical trial interim report. Vis. Res..

[10] Stingl K, Bartz-Schmidt K-U, Gekeler F, Kusnyerik A, Sachs H, Zrenner E (2013). Functional outcome in subretinal electronic implants depends on foveal eccentricity. Investig. Opthalmol. Vis. Sci..

[11] Zrenner E (2002). Will retinal implants restore vision?. Science.

[12] Zrenner E, Bartz-Schmidt KU, Benav H, Besch D, Bruckmann A, Gabel V-P, Gekeler F, Greppmaier U, Harscher A, Kibbel S, Koch J, Kusnyerik A, Peters T, Stingl K, Sachs H, Stett A, Szurman P, Wilhelm B, Wilke R (2011). Subretinal electronic chips allow blind patients to read letters and combine them to words. Proc. R. Soc. B Biol. Sci..

[13] Zrenner E, Bartz-Schmidt KU, Besch D, Gekeler F, Koitschev A, Sachs HG, Stingl K, Gabel VP (2017). The subretinal implant ALPHA: implantation and functional results. Artificial Vision.

[14] Ahuja AK, Dorn JD, Caspi A, McMahon MJ, Dagnelie G, daCruz L, Stanga P, Humayun MS, Greenberg RJ, Argus II Study Group (2011). Blind subjects implanted with the Argus II retinal prosthesis are able to improve performance in a spatial-motor task. Br. J. Ophthalmol..

[15] Caspi A, Roy A, Wuyyuru V, Rosendall PE, Harper JW, Katyal KD (2018). Eye movement control in the Argus II retinal-prosthesis enables reduced head movement and better localization precision. Invest. Ophthalmol. Vis. Sci..

[16] Fernández E, Normann RA, Gabel VP (2017). CORTIVIS approach for an intracortical visual prostheses. Artificial Vision.

[17] Hornig R, Dapper M, Le Joliff E, Hill R, Ishaque K, Posch C, Benosman R, LeMer Y, Sahel J-A, Picaud S, Gabel VP (2017). Pixium vision: first clinical results and innovative developments. Artificial Vision.

[18] Kelly, S. K., Shire, D. B., Chen, J., Gingerich, M. D., Cogan, S. F., Drohan, W. A., Ellersick, W., Krishnan, A., Behan, S., Wyatt, J. L., & Rizzo, J. F. (2013). Developments on the Boston 256-channel retinal implant. In *2013 IEEE International Conference on Multimedia and Expo Workshops (ICMEW)*, 1–6. 10.1109/ICMEW.2013.6618445

[19] Menzel-Severing J, Laube T, Brockmann C, Bornfeld N, Mokwa W, Mazinani B, Walter P, Roessler G (2012). Implantation and explantation of an active epiretinal visual prosthesis: 2-year follow-up data from the EPIRET3 prospective clinical trial. Eye.

[20] Shivdasani MN, Luu CD, Cicione R, Fallon JB, Allen PJ, Leuenberger J, Suaning GJ, Lovell NH, Shepherd RK, Williams CE (2010). Evaluation of stimulus parameters and electrode geometry for an effective suprachoroidal retinal prosthesis. J. Neural Eng..

[21] Lewis PM, Ackland HM, Lowery AJ, Rosenfeld JV (2015). Restoration of vision in blind individuals using bionic devices: a review with a focus on cortical visual prostheses. Brain Res..

[22] Yue L, Weiland JD, Roska B, Humayun MS (2016). Retinal stimulation strategies to restore vision: fundamentals and systems. Prog. Retin. Eye Res..

[23] Paraskevoudi N, Pezaris JS (2019). Eye movement compensation and spatial updating in visual prosthetics: mechanisms, limitations and future directions. Front. Syst. Neurosci..

[24] Burr D (2004). Eye movements: keeping vision stable. Curr. Biol..

[25] Inaba N, Kawano K (2016). Eye position effects on the remapped memory trace of visual motion in cortical area MST. Sci. Rep..

[26] Klier EM, Angelaki DE (2008). Spatial updating and the maintenance of visual constancy. Neuroscience.

[27] Rao HM, Mayo JP, Sommer MA (2016). Circuits for presaccadic visual remapping. J. Neurophysiol..

[28] Brickner RM (1936). Oscillopsia: a new symptom commonly occurring in multiple sclerosis. Arch. Neurol. Psychiatry.

[29] Gresty MA, Hess K, Leech J (1977). Disorders of the vestibulo-ocular reflex producing oscillopsia and mechanisms compensating for loss of labyrinthine function. Brain.

[30] Evans N (1989). The significance of nystagmus. Eye.

[31] Hafed ZM, Krauzlis RJ (2010). Microsaccadic suppression of visual bursts in the primate superior colliculus. J. Neurosci..

[32] Hafed ZM, Stingl K, Bartz-Schmidt K-U, Gekeler F, Zrenner E (2016). Oculomotor behavior of blind patients seeing with a subretinal visual implant. Vis. Res..

[33] Leopold DA, Logothetis NK (1998). Microsaccades differentially modulate neural activity in the striate and extrastriate visual cortex. Exp. Brain Res..

[34] Kagan I, Hafed ZM (2013). Active vision: microsaccades direct the eye to where it matters most. Curr. Biol..

[35] Coppola D, Purves D (1996). The extraordinarily rapid disappearance of entopic images. Proc. Natl. Acad. Sci..

[36] Brindley GS, Lewin WS (1968). The sensations produced by electrical stimulation of the visual cortex. J. Physiol..

[37] Bradley DC, Troyk PR, Berg JA, Bak M, Cogan S, Erickson R, Kufta C, Mascaro M, McCreery D, Schmidt EM, Towle VL, Xu H (2005). Visuotopic mapping through a multichannel stimulating implant in primate V1. J. Neurophysiol..

[38] Davis TS, Parker RA, House PA, Bagley E, Wendelken S, Normann RA, Greger B (2012). Spatial and temporal characteristics of V1 microstimulation during chronic implantation of a microelectrode array in a behaving macaque. J. Neural Eng..

[39] Dobelle WH, Mladejovsky MG (1974). Phosphenes produced by electrical stimulation of human occipital cortex, and their application to the development of a prosthesis for the blind. J. Physiol..

[40] Pezaris JS, Reid RC (2007). Demonstration of artificial visual percepts generated through thalamic microstimulation. Proc. Natl. Acad. Sci..

[41] Schmidt EM, Bak MJ, Hambrecht FT, Kufta CV, O’Rourke DK, Vallabhanath P (1996). Feasibility of a visual prosthesis for the blind based on intracortical micro stimulation of the visual cortex. Brain.

[42] Sinclair NC, Shivdasani MN, Perera T, Gillespie LN, McDermott HJ, Ayton LN, Blamey PJ, for the Bionic Vision Australia Consortium (2016). The appearance of phosphenes elicited using a suprachoroidal retinal prosthesis. Investig. Opthalmol. Vis. Sci..

[43] Stronks HC, Dagnelie G, Dagnelie G (2011). Phosphene mapping techniques for visual prostheses. Visual Prosthetics.

[44] Tehovnik EJ, Slocum WM (2007). Phosphene induction by microstimulation of macaque V1. Brain Res. Rev..

[45] Veraart C, Raftopoulos C, Mortimer JT, Delbeke J, Pins D, Michaux G, Vanlierde A, Parrini S, Wanet-Defalque M-C (1998). Visual sensations produced by optic nerve stimulation using an implanted self-sizing spiral cuff electrode. Brain Res..

[46] Erickson-Davis C, Korzybska H (2021). What do blind people “see” with retinal prostheses? Observations and qualitative reports of epiretinal implant users. PLoS ONE.

[47] Brady-Simmons C, Van Der Biest R, Bozeman L (2016). Miami lighthouse for the blind and visually impaired case study: vision rehabilitation for the first Florida resident to receive the Argus II “bionic eye”. J. Vis. Impair. Blind..

[48] Sabbah N, Authie CN, Sanda N, Mohand-Said S, Sahel J-A, Safran AB (2014). Importance of eye position on spatial localization in blind subjects wearing an Argus II retinal prosthesis. Invest. Ophthalmol. Vis. Sci..

[49] Prabhu D, Wise L, MacMahon C, De Man M, Petoe M, McCarthy C (2021). Effect of camera position on egocentric localisation with simulated prosthetic vision. Eng. Res. Express.

[50] Titchener SA, Kvansakul J, Shivdasani MN, Fallon JB, Nayagam DAX, Epp SB, Williams CE, Barnes N, Kentler WG, Kolic M, Baglin EK, Ayton LN, Abbott CJ, Luu CD, Allen PJ, Petoe MA (2020). Oculomotor responses to dynamic stimuli in a 44-channel suprachoroidal retinal prosthesis. Transl. Vis. Sci. Technol..

[51] Bourkiza B, Vurro M, Jeffries A, Pezaris JS (2013). Visual acuity of simulated thalamic visual prostheses in normally sighted humans. PLoS ONE.

[52] McIntosh, B. P. (2015). *Intraocular and extraocular cameras for retinal prostheses: effects of foveation by means of visual prosthesis simulation*. http://digitallibrary.usc.edu/cdm/ref/collection/p15799coll3/id/530967. Acccessed 22 July 2020.

[53] Rassia KEK, Pezaris JS (2018). Improvement in reading performance through training with simulated thalamic visual prostheses. Sci. Rep..

[54] Titchener SA, Shivdasani MN, Fallon JB, Petoe MA (2018). Gaze Compensation as a technique for improving hand-eye coordination in prosthetic vision. Transl. Vis. Sci. Technol..

[55] Vurro M, Crowell AM, Pezaris JS (2014). Simulation of thalamic prosthetic vision: reading accuracy, speed, and acuity in sighted humans. Front. Hum. Neurosci..

[56] da Cruz L, Coley BF, Dorn J, Merlini F, Filley E, Christopher P, Chen FK, Wuyyuru V, Sahel J, Stanga P, Humayun M, Greenberg RJ, Dagnelie G, for the Argus II Study Group (2013). The Argus II epiretinal prosthesis system allows letter and word reading and long-term function in patients with profound vision loss. Br. J. Ophthalmol..

[57] Mansfield JS, Ahn SJ, Legge GE, Luebker A (1993). A new reading-acuity chart for normal and low vision. Ophthalmic Vis. Opt. Noninvasive Assess. Vis. Syst. Tech. Dig..

[58] Crossland MD, Legge GE, Dakin SC (2008). The development of an automated sentence generator for the assessment of reading speed. Behav. Brain Funct..

[59] Stronks HC, Dagnelie G (2014). The functional performance of the Argus II retinal prosthesis. Expert Rev. Med. Devices.

[60] Sommerhalder J, Rappaz B, de Haller R, Fornos AP, Safran AB, Pelizzone M (2004). Simulation of artificial vision: II. Eccentric reading of full-page text and the learning of this task. Vis. Res..

[61] Gilchrist ID, Brown V, Findlay JM (1997). Saccades without eye movements. Nature.

[62] Barry MP, Dagnelie G (2016). Hand-camera coordination varies over time in users of the Argus II retinal prosthesis system. Front. Syst. Neurosci..

[63] Killian NJ, Vurro M, Keith SB, Kyada MJ, Pezaris JS (2016). Perceptual learning in a non-human primate model of artificial vision. Sci. Rep..

[64] Cehajic Kapetanovic J, Troelenberg N, Edwards TL, Xue K, Ramsden JD, Stett A, Zrenner E, MacLaren RE (2020). Highest reported visual acuity after electronic retinal implantation. Acta Ophthalmol..

[65] Garcia S, Petrini K, Rubin GS, Da Cruz L, Nardini M (2015). Visual and non-visual navigation in blind patients with a retinal prosthesis. PLoS ONE.

[66] Sommerhalder J, Pérez-Fornos A, Gabel VP (2017). Prospects and limitations of spatial resolution. Artificial Vision.

[67] Pezaris JS, Reid RC (2009). Simulations of electrode placement for a thalamic visual prosthesis. IEEE Trans. Biomed. Eng..

[68] Stingl K, Schippert R, Bartz-Schmidt KU, Besch D, Cottriall CL, Edwards TL, Gekeler F, Greppmaier U, Kiel K, Koitschev A, Kühlewein L, MacLaren RE, Ramsden JD, Roider J, Rothermel A, Sachs H, Schröder GS, Tode J, Troelenberg N, Zrenner E (2017). Interim results of a multicenter trial with the new electronic subretinal implant Alpha AMS in 15 patients blind from inherited retinal degenerations. Front. Neurosci..

[69] Humayun MS, Dorn JD, da Cruz L, Dagnelie G, Sahel J-A, Stanga PE, Cideciyan AV, Duncan JL, Eliott D, Filley E, Ho AC, Santos A, Safran AB, Arditi A, Del Priore LV, Greenberg RJ (2012). Interim results from the international trial of second sight’s visual prosthesis. Ophthalmology.

[70] Ramanauskas N (2006). Calibration of video-oculographical eye-tracking system. Elektronika Ir Elektrotechnika.

[71] Zhu, Z., Ji, Q., & Bennett, K. P. (2006). Nonlinear eye gaze mapping function estimation via support vector regression. In *18th International Conference on Pattern Recognition (ICPR’06)*, 1132–1135. 10.1109/ICPR.2006.864

[72] Barsingerhorn AD, Boonstra FN, Goossens J (2018). Development and validation of a high-speed stereoscopic eyetracker. Behav. Res. Methods.

[73] Dobelle WH, Turkel J, Henderson DC, Evans JR (1979). Mapping the representation of the visual field by electrical stimulation of human visual cortex. Am. J. Ophthalmol..

[74] Everitt BS, Rushton DN (1978). A method for plotting the optimum positions of an array of cortical electrical phosphenes. Biometrics.

[75] Kar A, Corcoran P (2017). A review and analysis of eye-gaze estimation systems, algorithms and performance evaluation methods in consumer platforms. IEEE Access.

